# The effects of exercise and active assisted cycle ergometry in post-operative total knee arthroplasty patients - a randomized controlled trial

**DOI:** 10.1186/s40634-021-00363-w

**Published:** 2021-06-22

**Authors:** P. Sanzo, S. Niccoli, K. Droll, D. Puskas, C. Cullinan, S. J. Lees

**Affiliations:** 1 School of Kinesiology, Lakehead University, 955 Oliver Rd, Thunder Bay, P7B 5E1 Canada; 2 Northern Ontario School of Medicine, 955 Oliver Rd, Thunder Bay, P7B 5E1 Canada; 3 Thunder Bay Regional Health Sciences Centre, 980 Oliver Rd, Thunder Bay, P7B 6V4 Canada

**Keywords:** Total knee replacement, Exercise, Rehabilitation

## Abstract

**Purpose:**

The purpose of this study was to examine the effect of the use of an active assisted cycle ergometer as an adjunct to post-operative treatment following total knee arthroplasty.

**Method:**

A total of 55 participants aged 50–80 years who had undergone unilateral total knee arthroplasty were randomly assigned to either the control group (standard of care) or the active assisted cycle ergometer (AACE) group. The effect on patient motivation, blood biomarkers, and knee pain, function, range of motion (ROM), strength, and swelling was examined. Qualitative feedback was also obtained post-operatively.

**Results:**

Although there was no statistically significant difference in the standard of care compared to the AACE group, there was a trend for a greater reduction in knee pain on the visual analog scale, improved Lower Extremity Functional Scale scores, and knee extension ROM and strength. A greater percentage of the experimental group demonstrated higher motivation. There was no significant difference in swelling or blood biomarker measures. Qualitative feedback from the AACE group post-operatively was also positive.

**Conclusions:**

The use of an AACE protocol as an adjunct to total knee arthroplasty rehabilitation may improve post-operative clinical outcomes. This study has been registered at clinicaltrials.gov (identifier NCT02265523, Oct 16 2014). Level of evidence: Level 1 – randomized controlled trial. Further research with a larger sample size is needed to confirm the benefits of the ergometer use.

**Supplementary Information:**

The online version contains supplementary material available at 10.1186/s40634-021-00363-w.

## Introduction

The combination of an increasing aging population, higher rates of osteoarthritis (OA), and higher rates of obesity have resulted in an increased incidence of total knee arthroplasty (TKA) [1–7]. Joint replacement surgery has been reported to be the most effective treatment for severe OA in reducing pain and disability [8, 9]. With all surgeries, however, one must also consider the possibility of negative effects and adverse events. Knee flexion contractures have been reported to be a risk factor for the development of a thromboembolic negative event and as a result, improved knee ROM must be maximized to reduce this possible adverse event [10]. Surgery is often combined with post-operative rehabilitation and treatment often includes education, active and passive exercises, and therapeutic modalities to maximize recovery and reduce post-operative complications [10, 11]. It is proposed that exercise reduces pain and improves function. Although the optimal exercise program has not been determined, it continues to be an important part of rehabilitation post-operatively. Factors that may affect the outcome following surgery also include psychological variables, self-motivation and compliance, the presence of comorbidities, gender, and age [12, 13]. With increased utilization rates in medical and rehabilitative care, and with the rising healthcare costs and budgetary constraints comes a time of fiscal accountability for healthcare providers and patients [5, 14, 15]. The current trend following surgical procedures is toward a reduction in the length of inpatient stays and early discharge from hospital to decrease the pressure on hospital costs and to demonstrate fiscal responsibility [11, 16]. As a result, it is imperative to find the optimal combination of treatments and exercise to assist with cost control, optimize and restore functional abilities, ROM and strength, minimize post-operative pain and adverse events, increase self-motivation, and at the same time insure patient compliance and satisfaction [17, 18]. Therefore, the purpose of this study was to: 1) determine the efficacy of two post-surgical exercise programs on knee pain, function, ROM, strength, and swelling (girth); 2) determine if the use of the active assisted cycle ergometer (AACE) had any impact on two blood biomarkers associated with risk of thrombogenic events; 3) examine the effect of, and improve patient compliance and motivation following TKA; and 4) capture the participants’ interpretation of their rehabilitation success and thoughts on the use of the AACE as an adjunct to treatment.

## Materials and methods

### Study information

The study design and reporting followed the Consolidated Standards of Reporting Trials [19]. Written informed consent was obtained before any functional tests, measurements, or intervention was performed or recorded. All plans and documents associated with the study were formally approved prior to participant enrolment by the research ethics board and the Clinical Research Services Department at the Thunder Bay Regional Health Sciences Centre as well as the research ethics board at Lakehead University. The randomized controlled trial was also registered in the public repository at clinicaltrials.gov (identifier NCT02265523).

### Recruitment, screening, and enrollment

Participants awaiting unilateral TKA were recruited from the wait list at the local acute care hospital Thunder Bay Regional Health Sciences Centre and orthopedic surgeons’ clinical practice Thunder Bay Regional Health Sciences Centre. Prospective male and female participants were screened using the following inclusion/exclusion criteria:

Inclusion criteria.

1) 50–80 years of age;

2) willing to provide informed consent; and.

3) willing to be randomized to either of the post-operative treatment pathways and willing to follow the study protocol.

Exclusion criteria.

1) presence of serious cardiac, renal, hepatic, neoplastic, or psychiatric diseases;

2) presence of diabetes; and.

3) abnormal thyroid and adrenal function.

Initial target sample size was 100 participants total with 50 per group and was calculated based on a power analysis performed on ROM measures as this measurement would likely result in the highest variability. It was determined that there could be a 10% change +/− 15% in ROM between both groups which would result in a power of 0.928 for a group size of 50. To achieve a power of 0.8, the minimum group size is 35 participants. This trial was stopped before reaching the targeted recruitment number as the research team reached their scheduled date of closure, resulting in the end of funding.

After receiving written informed consent, participants were assigned to either the control group or the AACE group by the factors of age and sex using QMinim (http://rct.mui.ac.ir/qminim/index.php), a program which performs restricted randomization using a biased coin probability method (base probability of 0.7). Demographic information was obtained (height, mass, age, and sex), as well as information related to past medical history and past surgical history of the knee. All data was de-identified prior to publishing. The control group participants were directed by the physiotherapists to follow a standard post-operative exercise protocol (Supplementary Information) only through the duration of the study, while AACE group participants were directed to follow that same standard post-operative exercise protocol with the addition of the Viscus© (AACE) use (Andre Riopel, Sault Ste. Marie, Canada). The AACE has a smooth mechanism allowing movement with minimal effort, assisted with a flywheel, while working to increase ROM. These participants were directed to use the AACE daily in a progressive manner for 6–12 weeks (Supplementary Table 1) and record their actual use in minutes.

### Clinical measurements

Measurements were taken at rest pre-operatively and 6–12 weeks post-operatively. Self-reported measures of knee pain and function were measured using the Visual Analog Scale (VAS) [17, 20, 21] and the Lower Extremity Functional Scale (LEFS) [22]. Knee swelling was measured via girth measurements taken at 15 cm superior to the superior pole of the patella, at the supra- and infrapatellar regions, and 15 cm inferior to the inferior pole of patella. Knee flexion and extension ROM were measured using a goniometer [23, 24]. Motivation was measured using the Behavioural Regulation in Exercise Questionnaire (BREQ2) [25] and resisted isometric knee flexion and extension strength was measured using a Lafayette Manual Muscle Tester model 01165 [23, 26].

### Blood collection and biomarker analysis

Blood draws occurred pre-operatively, 2 days post-operatively, and 6–12 weeks post-operatively in sodium citrate tubes and were further processed to extract plasma. Plasma was analysed for amounts of Interleukin-10 (IL-10, using R&D Systems cat. #HS100C) and P-selectin (using Abcam cat. #100631) with Enzyme-Linked Immunosorbent Assays (ELISAs) as per the manufacturer’s protocol.

### Participant satisfaction survey

An optional survey was offered to all participants at the end of the study. This survey was designed to capture the participants’ interpretation of their rehabilitation success, as well as their thoughts on whether they found the AACE helpful in their progression. This survey was created by the research team.

### Data analysis

Data are presented as mean (standard deviation (SD)). For demographic information, comparisons were made using two-way analysis of variance (ANOVA). For ROM, strength, LEFS, VAS, knee swelling, BREQ2, and blood biomarkers comparisons were made using a repeated measures two-way ANOVA. These were followed by Fisher’s LSD post-hoc tests (GraphPad Prism, San Diego, CA, USA). For LEFS, VAS, and BREQ2 a Chi-Square distribution was also performed on the proportion of participants that reported a clinically relevant improvement. Significance was accepted at *P* ≤ 0.05.

## Results

### Demographic information

**Fig. 1:**
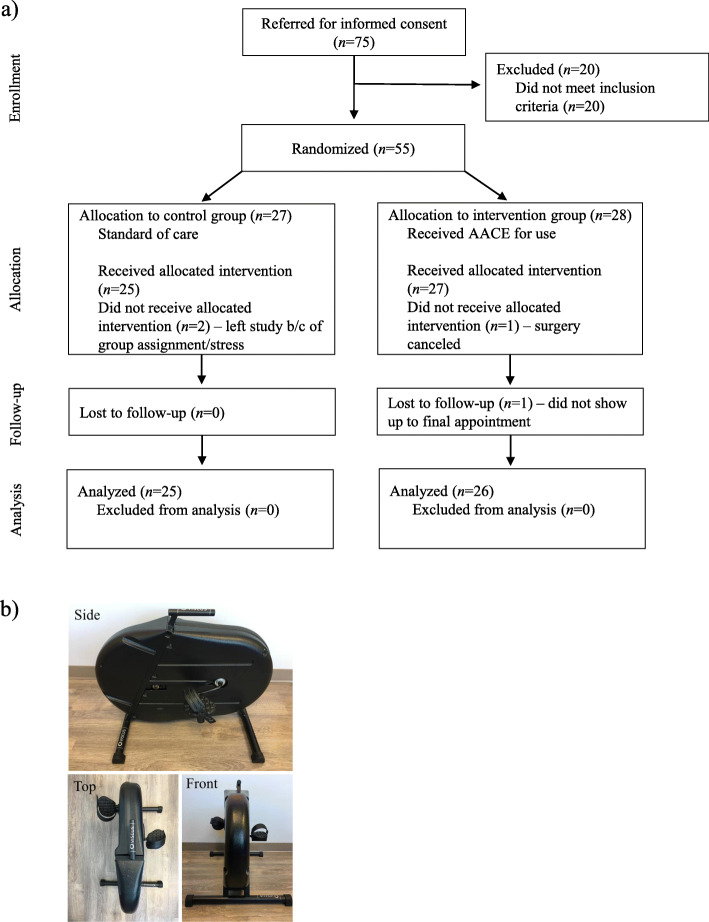
Study Details. **A**) Consolidated Standards of Reporting Trials (CONSORT) flow diagram of the progress through the trial. **B**) Viscus© active assisted cycle ergometer (AACE)

A total of 55 participants were enrolled in the study, and randomly assigned to either group. The participant numbers for enrolment, allocation, follow-up, and analysis are outlined in Fig. 1, along with a picture of the AACE. The demographic information for all enrolled participants is shown in Table 1, with the exception of one participant who withdrew before any data was collected. One study participant in the AACE group experienced increased knee swelling of which the physician determined was normal at the 6-week post-op mark, however, the participant was advised to decrease use of the AACE until swelling subsided. The mean age of participants was 63.9 years in the control group and 63.5 years in the AACE group, with a range of 50–80 years. The mean follow-up time between post-operative day 2 and the final measures appointment was 7 weeks. There was no significant difference in follow up times between groups (*P* = .14; 95% CI [− 2.5, 16.9]).

**Table 1:** Demographic information. Information presented as average (SD)

	Control			Active Assisted Cycle Ergometer		
	Males (*n* = 10)	Females (*n* = 16)	Combined (*n* = 26)	Males (*n* = 13)	Females (*n* = 15)	Combined (*n* = 28)
Age in years	64.50 (4.62)	63.56 (9.14)	63.92 (7.62)	66.15 (8.25)	61.20 (5.37)	63.50 (7.18)
Height in cm	179.31 (9.58)	159.31 (4.88)	167.00 (12.07)	175.05 (6.01)	161.94 (8.02)	168.03 (9.68)
Weight in kg	103.00 (13.85)	85.41 (18.44)	92.17 (18.69)	97.85 (17.21)	95.00 (20.26)	96.32 (18.62)
Total number of days in intervention stage of study		*n* = 24	45.88 (11.02)		*n* = 26	53.04 (21.10)

SD = standard deviation

### Clinical measurements

**Fig. 2:**
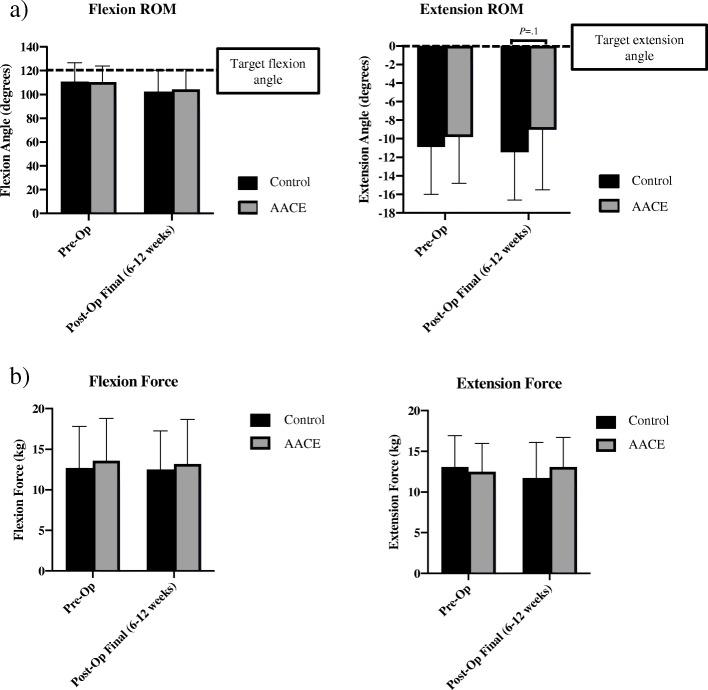
Measured Knee Function. **A**) Goniometric range of motion (ROM) pre-op and post-op measures for knee flexion and extension in the control and active assisted cycle ergometer (AACE) groups. *n* = 25–26 per group. **B**) Resisted isometric knee flexor and extensor strength using a Lafayette Manual Muscle Tester pre-op and post-op measures for the control and active assisted cycle ergometer (AACE) groups. *n* = 24–26 per group

Neither the control nor the AACE group demonstrated significant improvements in ROM measures (Fig. 2A) or strength measures (Fig. 2B) over the course of the study, however, while not statistically significant, the AACE group demonstrated a trend towards an improvement in extension angle compared to the control group (*P* = .1; 95% CI [− 5.15, 0.45]).

**Fig. 3:**
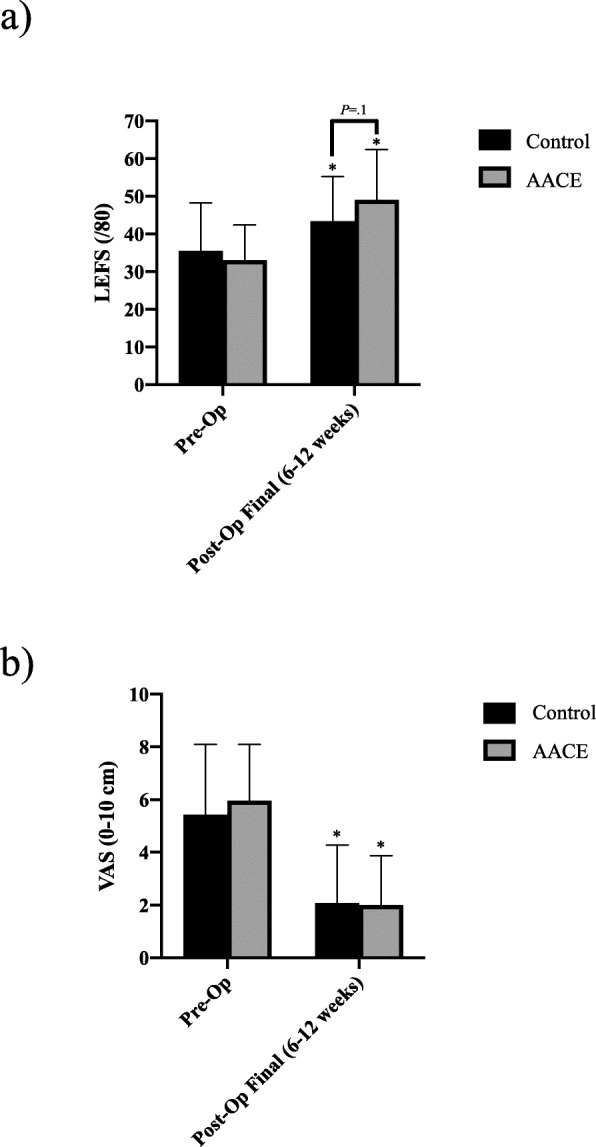
Self-Reported Knee Function. **A**) Lower extremity functional scale score for pre-op and post-op measures in the control and active assisted cycle ergometer (AACE) groups. * denotes significant difference from corresponding pre-operative measures. *n* = 24–26 per group. **B**) Visual analog pain pre-op and post-op measures in the control and active assisted cycle ergometer (AACE) groups. * denotes significant difference from corresponding pre-operative measures. *n* = 25–26 per group

Both the control group and the AACE group demonstrated statistically significant improvements in LEFS score (Fig. 3A) post-operatively compared to pre-operative measures (control: *P* = .01 95% CI [− 14.1, − 1.8], AACE: *P <* .001 95% CI [− 22, − 10.1]). While it is not statistically significant, the AACE group demonstrated a trend towards a higher functional score than the control group (*P* = .1 95% CI [− 11.9, 1]). It is accepted that an increase of at least 9 points in the LEFS score represents clinical improvements in lower extremity function [27]. There was a non-significant difference between the percentage of the participants in the AACE group who had clinically significant improvements in function (73%, 19 out of 26) compared to the control group (67%, 16 out of 24).

Both the control group and the AACE group demonstrated statistically significant improvements in pain measures post-operatively compared to pre-operative measures (control: *P* < .001 95% CI [2.18, 4.45], AACE: *P* < .001 95% CI [2.83, 5.07]) (Fig. 3B). It is accepted that a decrease of at least 3 cm on the VAS represents clinical improvements in pain [28]. There was a non-significant difference between the percentage of participants in the AACE group who demonstrated clinically significant improvements in pain (69%, 18 out of 26) compared to the control group (60%, 15 out of 25). Neither the control group nor the AACE group demonstrated any significant changes in swelling measures (Supplementary Table 2).

With respect to motivation, the BREQ2 analysis revealed that 50% (12 out of 24) of the control group participants had improved scores, while 58% (15 out of 26) of the AACE group participants had improved scores (Table 2). A higher final score compared to initial score represents an improvement in greater relative autonomy (self-determination) and motivation. The final scores were calculated from the following subsections: Amotivation, External Regulation, Introjected Regulation, Identified Regulation, and Intrinsic Regulation.

**Table 2:** BREQ2 pre-op and post-op measures. Results presented as average (SD). *n* = 24–26 per group

Group	Time Point	Amotivation	External Regulation	Introjected Regulation	Identified Regulation	Intrinsic Regulation	Total
Control	Pre-Operative	−0.72 (1.59)	− 1.38 (2.23)	− 2.04 (1.09)	6.46 (1.49)	8.95 (3.03)	11.28 (6.04)
	Post-Operative	−0.20 (0.57)	−1.06 (1.66)	− 1.93 (1.26)	6.38 (1.17)	8.31 (2.43)	11.49 (4.40)
						% Improved	50
Active Assisted Cycle	Pre-Operative	−0.40 (1.00)	−0.85 (1.45)	−1.36 (1.04)	5.79 (2.15)	8.08 (3.13)	11.22 (5.42)
	Post-Operative	−0.20 (0.75)	−0.60 (1.12)	−1.46 (0.93)	6.35 (1.78)	8.08 (2.64)	12.16 (4.72)
Ergometer						% Improved	57.69

SD = standard deviation

**Fig. 4:**
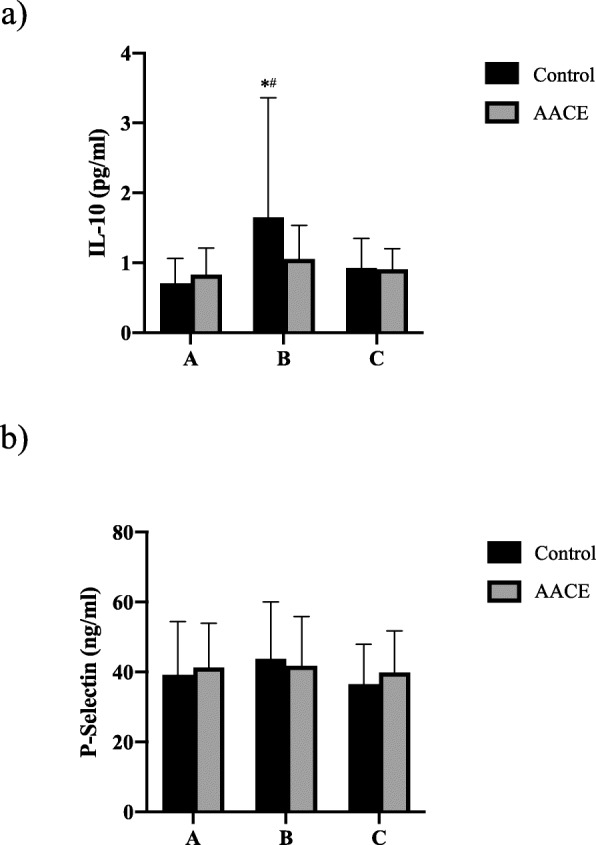
Blood Biomarkers. **A**) Plasma IL-10 concentration pre-op and post-op measures in the control and active assisted cycle ergometer (AACE) groups. A = pre-op, B = 2 days post-op, C = 6–12 weeks post-op. * denotes significant difference from corresponding pre-operative measures (*P <* .001 95% CI [− 1.372, − 0.523]), # denotes significant difference from corresponding 6–12 week post-operative measures (*P* = .001 95% CI [0.299, 1.149]). *n* = 21–25 per group; only participants with all three blood draws were analyzed. **B**) Plasma P-selectin concentration pre-op and post-op measures in the control and active assisted cycle ergometer (AACE) groups. A = pre-op, B = 2 days post-op, C = 6–12 weeks post-op. *n* = 21–25 per group; only participants with all three blood draws were analyzed

There were no statistically significant differences in plasma levels of IL-10 or P-selectin between the control group and the AACE group (Fig. 4).

### Participant satisfaction survey

In total, 21 study participants from the control group and 23 study participants from the AACE group completed the participant satisfaction survey (Supplementary Information). Most participants in the AACE group reported being satisfied with their improvement in ROM (96% of participants, 22 out of 23) and joint function (100% of participants, 23 out of 23) compared to participants in the control group (81%, 17 out of 21 and 90%, 19 out of 21, respectively). Additionally, it was reported that 91% (20 out of 22) of the participants enjoyed using the AACE and 82% (18 out of 22) thought that it helped improve their rehabilitation success. In order to work towards improving the study design and success in the future, participants in the AACE group were asked directly about their use of the unit. Most participants (82%, 18 out of 22) felt that they would be comfortable pushing themselves further while using the unit, while only 59% (13 out of 22) of them thought they would benefit from longer use of the unit with more instructions during use (50%, 11 out of 22).

## Discussion

The aim of this study was to examine the effect of the use of an active assisted cycle ergometer as an adjunct to post-operative treatment following TKA. Measures of function, pain, and motivation were used to assess the success of this intervention.

The use of regular cycling as part of the rehabilitation process following total joint arthroplasty has resulted in mixed reviews. Positive effects have been reported post THA but not post TKA on self reported measures of physical function [29]. Suggested reasons for the differences have been attributed to possible increased pain and swelling in the TKA population with the introduction of this active exercise with the knee positioned below the heart. Similarly, mixed findings regarding the use of stationary cycling following TKA have been reported when seeking consensus among treating physiotherapists [30]**.** While active assisted exercises have been suggested, the use of active assisted cycling as an adjunct to treatment following surgery has not been part of the standard of care programs. The design of the AACE provides this combination of cycling with an active assist mechanism, aiming to allow patients to improve ROM while avoiding pain and swelling.

Improvement in ROM is one of the main goals of TKA [8]. It has been shown that ROM at intake and post-rehabilitation can predict the functional outcome, length of rehabilitation required, and ability to adjust in the hospital, home and community following TKA [31]. In the case of the current study, use of the AACE as part of the rehabilitation program led to extension angles closer to post-operative targets compared to the standard of care program alone. While this improvement was not statistically significant, its clinical significance is relevant and may predict more positive long-term outcomes that were not measured in this study. Similarly, there was no statistically significant improvement in knee flexor or extensor strength, but the trend was for higher force outputs in the AACE group. This trend towards an improvement in both ROM and strength following AACE use, if confirmed through further studies, could decrease the need for more intensive rehabilitative strategies and, therefore, work to control healthcare costs associated with TKA [11, 16].

While ROM and strength are important outcome measures, joint function during completion of everyday tasks and activities could be deemed just as important for patient satisfaction following TKA, as well as for successful rehabilitation. The self-reported LEFS scoring system was developed to assess this in particular [27]. The larger percentage of participants in the AACE group showing clinically significant improvements in their functional status suggests the addition of the AACE could improve quality of life for patients following TKA, beyond what standard of care rehabilitation could do.

Pain management is a principal aspect of recovery following TKA and is viewed as one of the top two reasons for dissatisfaction in surgical outcome alongside joint function [32]. While there was no significant difference in VAS measures post-operatively between the two groups, the AACE group had a larger percentage of participants who demonstrated clinically significant improvements in these pain scores [28]. This decrease in pain may have contributed to the overall trend of improvements in other clinical measures observed in this study. The importance of post-operative pain management has been reviewed extensively in the TKA population [18, 33] and there is currently no suggested standard intervention for the treatment of pain in the form of analgesic or opioid treatments. Therefore, if these results are confirmed through further studies, the use of this active assisted exercise as an intervention for pain could be promising.

There were no significant differences in the swelling and girth measures pre-operatively and post-operatively in both the standard of care (control group) and the AACE group. This demonstrated that use of the AACE did not have any negative consequences on swelling, an important finding as swelling is one of the reported negative outcomes resulting from post-operative cycling [29].

Two biomarkers of thrombogenic risk were measured in the present study. The first, P-selectin was chosen based on its involvement in the thrombogenic process. P-selectin is elevated in deep vein thrombosis [34] and useful in the diagnosis of deep vein thrombosis [35]. In addition, P-selectin has been shown to be associated with increased risk of arterial and venous thromboembolism in patients with [36, 37] and without cancer, [38] and with portal vein thrombosis in patients with cirrhosis [39]. The second, IL-10, was chosen based on evidence that it may be associated with decreased risk [40, 41] and perhaps may even have a protective effect on deep vein thrombosis [42]. The link between IL-10 and thrombosis is based on its anti-inflammatory properties. IL-10 is an anti-inflammatory cytokine and it has been shown to reduce inflammation in the venous wall [43–46]. In addition, IL-10 has been shown to have anticoagulant properties [47]. No significant differences were seen, however, with the blood biomarker analysis demonstrating that the use of such a device as an adjunct to treatment will not likely negatively impact swelling and delay progress in reaching the clinical and functional goals post-operatively.

Along with physical measures to assess the success of TKA, it has been reported that psychological health and motivation may also impact surgical outcome and, therefore, need to be addressed both pre- and post-operatively [48]. Our findings demonstrated that participants who used the AACE demonstrated a higher level of motivation which may have supported the trend of improved clinical outcomes.

### Limitations

The sample size and power of the current study may have been limiting. The ROM outcomes are highly variable relative to the magnitude of change during recovery. This is particularly evident in the knee extension ROM. The target ROM is 0 degrees, therefore, detecting statistically significant differences may be challenging and clinically meaningful differences may not always coincide with the statistical findings. Some additional limitations to consider in the current study include the lack of direct supervision during use of the AACE which may have hindered the overall progress and ability to achieve maximal improvements. The researchers did attempt to monitor compliance but the data is unclear and a better way to assess and monitor this should be considered in the future if the implementation of such a device is planned. The provision of instructions on how to readjust and optimally position the cycle ergometer closer to the participant especially as increased knee ROM was achieved so that the participant continued to cycle at the maximal knee flexion angle, for example, may have further improved the results. Either direct supervision or virtual follow up may be required to continue to progress the home exercises and use of the device, achieving even better results in the desired clinical measures. From a design perspective for the actual cycle ergometer, a new design of the pedals and crank mechanism by which the ability to enable the extension of the crank arms may have also allowed for increased ROM to be achieved early in the rehabilitation process. Lastly, longer follow-up times may be necessary to see if a bigger post-operative treatment effect occurred with the use of the AACE compared to the standard of care. This must be balanced with the need to know versus being fiscally responsible in the presence of positive clinical outcomes. Additionally, for the purpose of this study the research team was interested in outcomes immediately following AACE use rather than in the years after. It is also important to acknowledge that these short-term results can be indicative of longer-term follow-up findings as knee score measures involving pain, function, and ROM have been shown to remain statistically the same between 3 month and 1-year follow-up times, [49] and continue to remain relatively stable for as long as 10 years [50].

## Conclusions

The aim of this study was to examine the effect of the use of an active assisted cycle ergometer as an adjunct to post-operative treatment following TKA. Although there was not a statistically significant difference in the standard of care compared to the AACE group treatment protocol, there was a trend for a greater reduction in knee pain on the VAS and improved LEFS scores, and knee extension ROM and strength measures. The results of this study warrant further investigation into the use of an AACE in post-operative rehabilitation following TKA.

## Supplementary Information

**Additional file 1.**

**Additional file 2.**

**Additional file 3.**

## Abbreviations

ROM: Range of motion
BREQ2: Behavioural Regulation in Exercise Questionnaire
OA: Osteoarthritis
TKA: Total knee arthroplasty
AACE: active assisted cycle ergometer.
VAS: Visual Analog Scale
LEFS: Lower Extremity Functional Scale
IL-10: Interleukin 10
ELISA: Enzyme-linked immunosorbent assay
SD: Standard deviation
ANOVA: Analysis of variance
CI: Confidence interval
