# Supplementary material for: The effects of exercise and active assisted cycle ergometry in post-operative total knee arthroplasty patients - a randomized controlled trial
Source: J Exp Orthop. 2021 Jun 22;8:41. doi: 10.1186/s40634-021-00363-w (PMC8219820; doi:10.1186/s40634-021-00363-w)
Supplement: Supplementary file 1 — Additional file 1. [file 40634_2021_363_MOESM1_ESM.pdf]

Participant Satisfaction Survey Version May 2018
PI: XXBLINDEDXX

Participant Number: _______
Date Survey Completed: ______________________ (month, day, year)
Date of Follow-up Appointment: _____________________ (month, day, year)

**How satisfied are you with the following?**	**Completely Dissatisfied (-2)**	**Somewhat Dissatisfied (-1)**	**Neutral (0)**	**Somewhat Satisfied (+1)**	**Completely Satisfied (+2)**
Your rehabilitation rate					
Your improvement in range of motion					
Your improvement in joint function					
**Total Score (sum of numbers shown under column headings):**					

Complete the sections below if you were in the flywheel group.

	**Completely Disagree (-2)**	**Somewhat Disagree (-1)**	**Neutral (0)**	**Somewhat Agree (+1)**	**Completely Agree (+2)**
I enjoyed using the flywheel.					
The flywheel was easy to use.					
I believe using the flywheel improved my rehabilitation.					
**Total Score (sum of numbers shown under column headings):**					

The data collected from the following section will be used to direct future study development.

	**Completely Disagree**	**Somewhat Disagree**	**Neutral**	**Somewhat Agree**	**Completely Agree**
I would be comfortable pushing myself further while using the flywheel.					
I would benefit from longer use of the flywheel.					
I would benefit from further instruction during flywheel use.					
